# Analysis of the expression and prognostic significance of DDK complex in Hepatocarcinoma

**DOI:** 10.1186/s12885-022-10475-w

**Published:** 2023-01-06

**Authors:** Min Wang, Zu-Hua Qiu, Yu-Zhuo Wang, Bo Lian, Jing-Kun Bai, Yong-Jie Zhou, Hong-Jie Ji

**Affiliations:** 1 School of Life Science and Technology, Weifang Medical University, No.7166, Baotongxi Street, Weifang, 261053 China; 2 Laboratory of Liver Transplantation, West China Hospital, Sichuan University, No.1, Keyuan 4 Road, Chengdu, 610041 China

**Keywords:** DDK, DBF4, CDC7, Hepatocellular carcinoma, DNA replication

## Abstract

**Background:**

Hepatocellular carcinoma (HCC) remains one of the most common and lethal malignancies worldwide. Although DBF4-dependent kinase (DDK) complex composed of CDC7 kinase and its regulatory subunit DBF4 has been shown to be overexpressed in primary tumors and promotes tumor development, while its role and prognostic value in HCC remain largely unknown. In the present study, the expression of DBF4 and CDC7 and their relationship with clinical characteristics were comprehensively analyzed.

**Methods:**

The mRNA expression profiles of HCC and the corresponding clinical data of HCC patients were downloaded from TCGA and GEO databases, respectively. The differences in DBF4 and CDC7 expression in tumor tissues and adjacent normal tissues were analyzed. HCC-derived tissue microarray (TMA) was used to evaluate and score the expression of CDC7 by immunohistochemistry (IHC) staining. The Kaplan–Meier method and the Cox regression method were used to analyze the relationship between overall survival and clinical characteristics of the patients. Gene set enrichment analysis (GSEA) was used to analyze the pathway enrichment of DBF4 and CDC7.

**Results:**

DBF4 and CDC7 had similar expression patterns in HCC patients. Detailly, compared with adjacent tissues, both mRNA and protein of DBF4 and CDC7 were significantly higher in HCC, and their expression was positively correlated with AJCC_T stage, clinical stage and G stage (grade) of liver cancer patients, and higher DBF4 or CDC7 expression predicted a worse prognosis in HCC patients with shorter overall survival (OS), recurrence-free survival (RFS), disease-specific survival (DSS) and progress-free survival (PFS). Cox regression analysis suggested that both DBF4 and CDC7 were independent risk factors for the prognosis of HCC patients in TCGA dataset. GSEA suggested that both DBF4 and CDC7 were positively correlated with cell cycle and DNA replication. Finally, the prognostic value of CDC7 was furtherly confirmed by TMA-based IHC staining results.

**Conclusions:**

Our study showed that DDK complex was significantly increased in HCC. Both DBF4 and CDC7 may be potential diagnostic and prognostic markers for HCC, and high expression of DDK members predicts a worse prognosis in patients with HCC, which may be associated with high tumor cell proliferation rate.

**Supplementary Information:**

The online version contains supplementary material available at 10.1186/s12885-022-10475-w.

## Introduction

According to the statistics, as one of the most malignant and lethal tumors, hepatocellular carcinoma (HCC) accounts for the majority of primary liver cancers (75% ~ 85%) [1, 2]. Actually, approximately 910,000 new cases of primary liver cancer were diagnosed and caused 830,000 deaths globally in only 2020 worldwide. Unfortunately, the newly diagnosed liver cancer in China accounts for about half of all the cases (~ 410,000) [2]. Despite great efforts have been paid to improve HCC diagnosis and treatments, the achievements are far from satisfactory and the poor prognosis of HCC patients is predictable [3]. Therefore, exploring promising molecular targets that can serve as prognostic indicators for HCC patients remains a great challenge for humans, especially the Chinese.

**Fig. 1:**
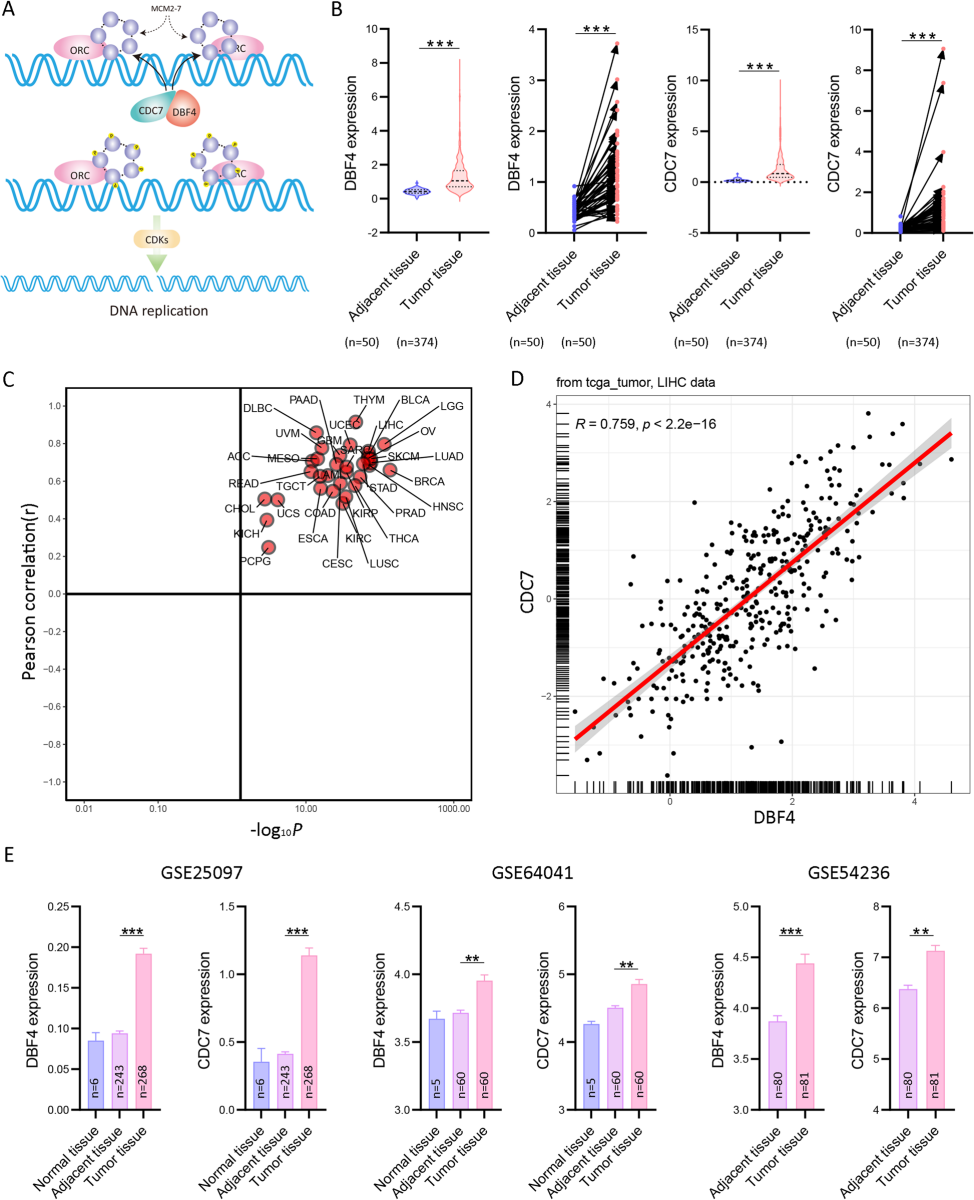
The expression of DDK complex in HCC. **A** The schematic diagram of DDK complex in DNA replication. **B** The mRNA level of DBF4 and CDC7 in TCGA HCC dataset. *** *P* < 0.001. **C** The pearson correlation between DBF4 and CDC7 in 26 types of cancers based on TCGA. **D** The pearson correlation of DBF4 and CDC7 in HCC dataset. **E** The expression of DBF4 and CDC7 in three GEO HCC datasets. ** *P* < 0.01, *** P < 0.001

Uncontrolled cell proliferation is one of the greatest features of all tumor cells, and there is abundant evidence that blocking cell proliferation or cell cycle processes has the potential to alleviate or cure tumors [4, 5]. DNA replication is pivotal to tumor cell proliferation and is a fundamental process. The DBF4-dependent kinase (DDK) composed of CDC7 kinase and its regulatory subunit DBF4, which is required for CDC7 kinase activity, is a critical regulator of DNA replication by catalyzing MCM helicase (MCM2-7) (Fig. 1A) [6, 7]. It has been reported that MCM family members can serve as prognostic biomarkers and MCM6 indicates adverse tumor features and poor outcomes and promotes S/G2 cell cycle progression in HCC [8–10]. And the other DNA replication regulator complex cyclin-dependent kinases (CDKs) have plenty of roles in HCC development and can serve as therapeutic targets [11–13]. While as the most critical regulator at DNA replication origin, the expression and potential role of DDK complex in HCC remain exclusive.

In this present study, we comprehensively analyzed the expression of DDK complex members (DBF4 and CDC7) and the relationship between their expression and clinical features based on HCC from our hospital and public databases (TCGA, GEO). The prognostic value of DDK complex members in HCC has been confirmed. Furtherly, the potential role of DDK complex members in cell cycle and DNA replication was further analyzed by Gene Set Enrichment Analysis (GSEA). These results may provide new clues for HCC precise diagnosis and therapy.

## Methods

### Data collection from public databases

Both the gene expression profiles of HCC patients and the corresponding clinical information were downloaded from the public TCGA HCC database (https:// portal.gdc.cancer.gov/repository) [14], in which 50 paired normal liver tissues and 374 HCC tissues were included. The mRNA expression of DDK complex members was downloaded from three GEO databases (GSE25097, GSE54236, GSE64041) (https://www.ncbi.nlm.nih.gov/geo/) [15–17]. All these public data were processed (downloaded, sorted and de-duplicated) by R studio software (R version 3.6.3, Boston, MA, USA). The normalized expression of DDK members was analyzed by GraphPad Prism 9.0 (GraphPad, San Diego, CA, USA).

### Kaplan–Meier plot for survival analysis

The survival analysis based on DDC complex expression and clinical information of HCC patients in TCGA HCC dataset was accomplished by online Web-Based Survival Analysis Tool (http://kmplot.com/analysis/). The best cutoff used to define low and high expression for each analyzed gene is generated as described [18]. Briefly, to find the best cutoff, they iterate over the input variable values from the lower quartile to the upper quartile and compute the Cox regression for each setting. The most significant cut-off value is used as the best cutoff to separate the input data into two groups.

### Gene set enrichment analysis (GSEA)

GSEA as a computational method is frequently used to determine whether an a priori defined set of genes shows statistically significant, concordant differences between two biological states. We firstly sorted out the genes that were negatively or positively correlated with our target genes by pearson correlation analysis based on TCGA HCC expression files, and GSEA was then used to identify the potential biological functions of target genes. In this study, GSEA was automatically carried out by the online website tool LinkedOmics (http://www.linkedomics.org/login.php) [19]. Genes highly correlated with DBF4 and CDC7 (*P* < 0.05) were subjected to further analysis (Enrichment analysis, KEGG pathway; Rank criteria, *P* value; Minimum number of genes, 3; Simulations, 1000). Detailly, 1000 gene set permutations were performed for each analysis. The normalized enrichment score (NES) was calculated and the significantly enriched gene sets were screened. Then, the enrichment pathways with a normal *p*-value < 0.05 and a false discovery rate (FDR) < 0.25 were selected.

### Patients, tissue microarray and IHC staining

A cohort of 110 patients with confirmed HCC underwent tumor resection at West China Hospital of Sichuan University between 2016 and 2017. OS was defined as the time between initial surgery and death. The paraffin-embedded tumor samples were made into tissue microarray (TMA) cores (1 mm diameter). Immunohistochemistry (IHC) staining was then performed on the TMA slides with CDC7 antibody (ab229187, Abcam, Cambridge, UK), and the results were interpreted by three pathologists blinded to clinical information. The expression level of CDC7 was scored according to the signal intensity and distribution as previously described [20]. Briefly, five 400 × -magnified areas were examined and assigned according to the following categories: 0, < 5%; 1, 5–25%; 2, 25–75%; 3, > 75%. The staining intensity was scored as follows: 1, weak; 2, moderate; and 3, intense. Tumor tissues with an IHC score of 0–3 were designated as low expression, and those with scores of 4–9 were designated as high expression. Classic core clinical features, such as age, AFP level, HBsAg, TNM stage, and tumor size, etc., were adopted to analyze the correlation of CDC7 with HCC. Approval for this study was granted by the Ethics Committee of the West China Hospital, Sichuan University, and each patient provided written informed consent.

### Western blot assay

Fresh frozen tissues from HCC patients were collected and lysed with RIPA buffer according to the manufacturer’s instructions (Beyotime Biotechnology, Shanghai, China). For each lane, 50 μg protein was loaded for detecting DBF4 and CDC7 on different gels. Proteins were separated with SDS-PAGE and transferred to 0.45 μm PVDF membranes. Membranes were then blocked with PBS containing 0.05% tween and 5% non-fat milk. DBF4 antibody (ab124707, Abcam, Cambridge, UK) and CDC7 antibody (ab229187, Abcam, Cambridge, UK) were used to detect the protein level of DBF4 and CDC7, and GAPDH (ET1601-4, HuaBio, Hangzhou, China) was used as an internal control.

### Statistical analysis

The DSS, PFS, RFS and OS of DDK complex members based on their expression and clinical data were automatically carried out by Kaplan–Meier Plotter (http://kmplot.com/analysis) [18]. All other statistical analyses were conducted by using the SPSS version 19.0 software program (SPSS Inc., Chicago, USA) or GraphPad Prism 7.0 (GraphPad, San Diego, CA, USA). The Kruskal–Wallis test, Wilcoxon rank test and logistic regression were used to analyze the relationship between DDK complex members and the clinical characteristics of HCC. Cox regression analysis was then used to compare the effects of CDC7 and clinical characteristics on OS and RFS. A *P* value of less than 0.05 was considered statistically significant.

## Results

### The expression status of DDK complex in HCC tissues

**Fig. 2:**
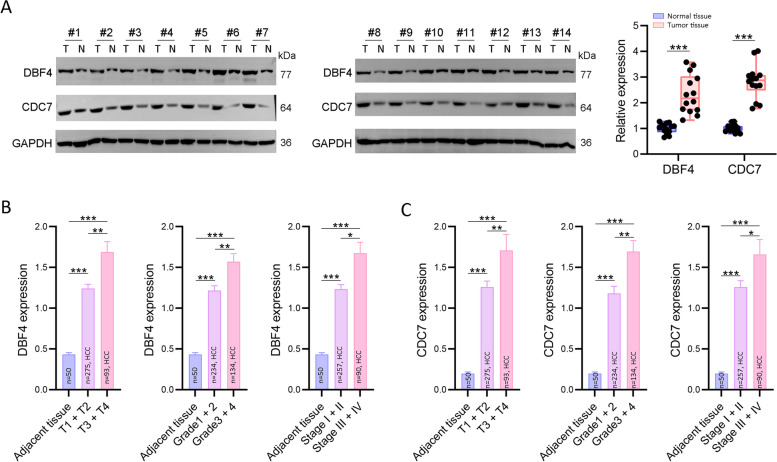
The association between the expression of DDK complex and clinical features of HCC patients. **A** The images and quantitative analysis of immunoblotting assay results of DBF4 and CDC7 (*n* = 14). ** *P* < 0.01. **B**, **C** The expression of DBF4 and CDC7 in different tumor T stages, grades and stages, * *P* < 0.05, ** *P* < 0.01, *** P < 0.001

To investigate the expression of DDK complex members in HCC patients, we firstly sorted out the expression profile of DBF4 and CDC7 in TCGA dataset, and we found both DBF4 and CDC7 were significantly highly expressed in HCC tumor tissues (Fig. 1B). Meanwhile, according to an online analysis platform (http://guotosky.vip:13838/GTBA/), we found DBF4 and CDC7 are highly correlated in all TCGA tumor types, especially in HCC (*R* = 7.459, *P* < 2.2e-16) (Fig. 1C, D). Additionally, we explored the expression of DBF4 and CDC7 from the expression profiles from GSE25097, GSE54236 and GSE64041, in which both of them were higher expressed in HCC tumor tissues compared with adjacent normal tissues and minimum expressed in healthy normal livers (Fig. 1E). Finally, the immunoblotting assay results further confirmed the elevated expression of DBF4 and CDC7 in HCC tissues (Fig. 2A).

### The DDK complex is highly associated with the clinical features of hepatocellular carcinoma patients

**Fig. 3:**
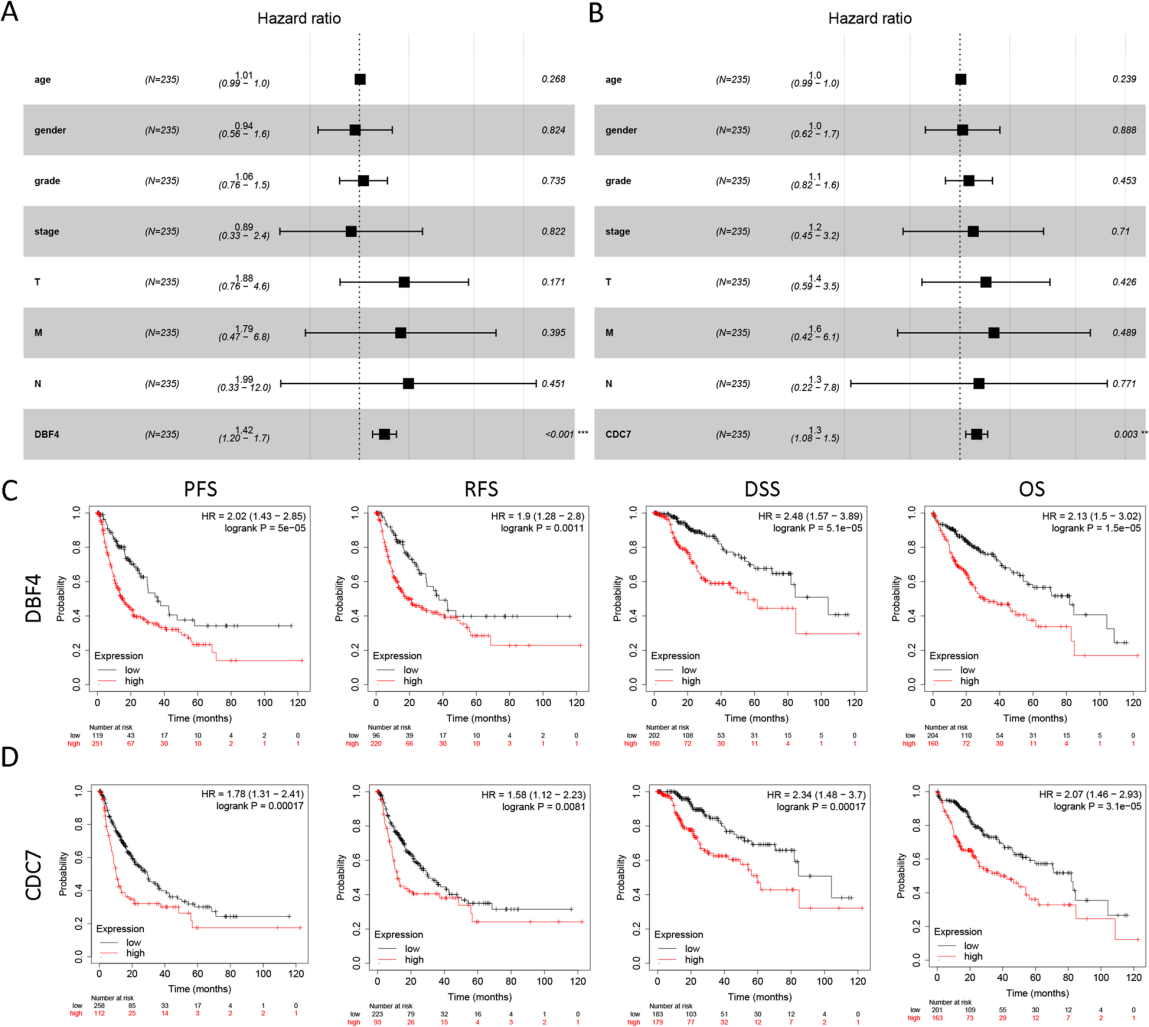
The prognostic value of DDK complex in TCGA dataset. **A**, **B** The multiCox proportional hazards regression analyses of DBF4 and CDC7. **C**, **D** The survival curves (PFS, RFS, DSS and OS) of TCGA HCC patients generated by Kaplan–Meier Plotter

Furtherly, we analyzed the expression of DDK complex members in different clinical statuses based on TCGA HCC dataset. As shown, the expression of DBF4 and CDC7 was positively correlated with tumor grade and stage, and was much higher in advanced HCC patients with higher clinical stage and grade (Fig. 2B, C). And both DBF4 and CDC7 were independent risk factors for HCC patients assessed by multiCox proportional hazards regression analyses (Fig. 3A, B). Additionally, according to Kaplan–Meier Plotter, HCC patients with higher DBF4 or CDC7 expression had worse OS, RFS and DSS (Fig. 3C, D).

### The prognostic value of CDC7 in HCC patients

**Fig. 4:**
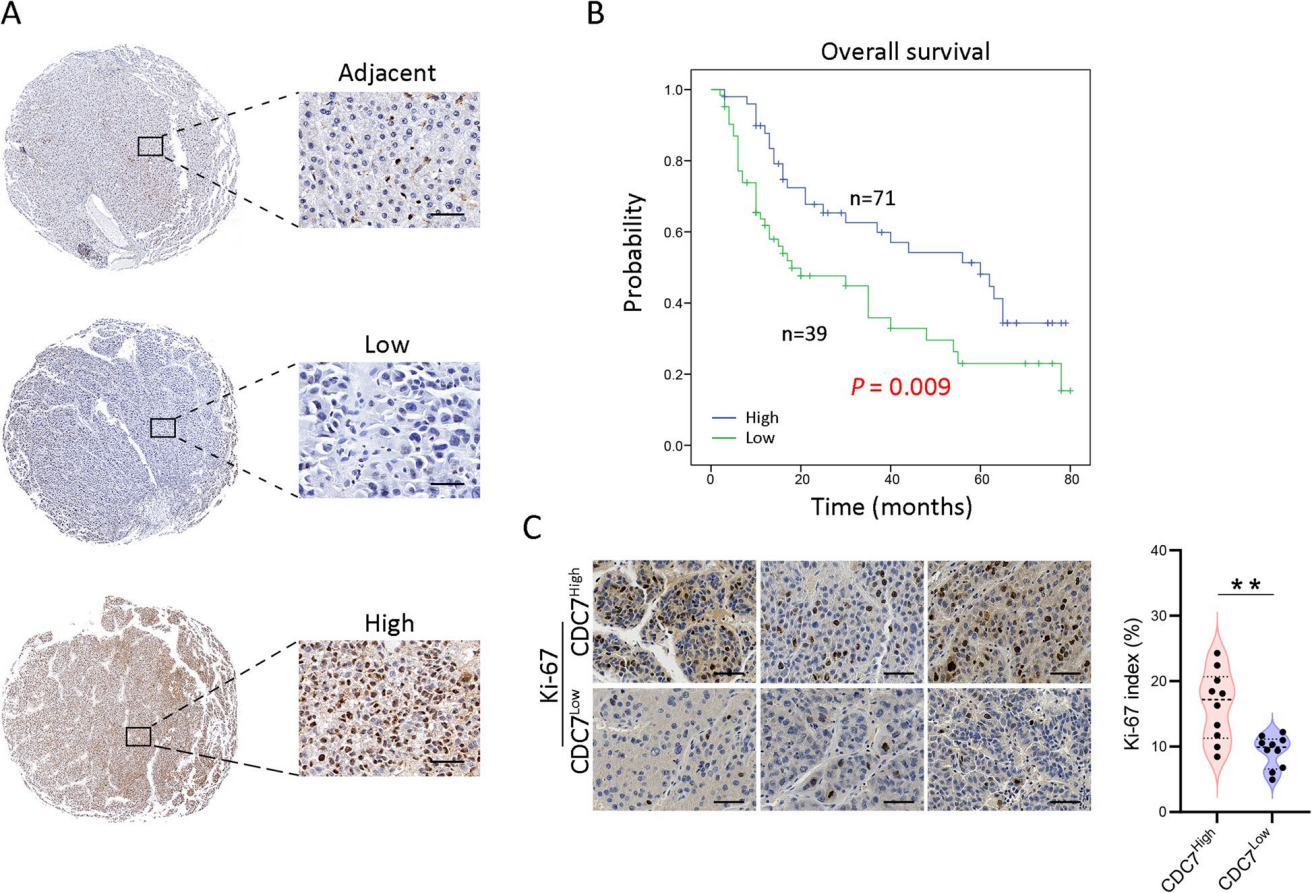
High expression of CDC7 indicates shorter OS and higher tumor cell proliferation. **A** The representative images of CDC7 staining in adjacent and HCC tumor tissues. Scale bar, 50 μm. **B** The OS curve of HCC patients based on CDC7 IHC scores. **C** The representative images and positive rate of Ki-67 staining results (*n* = 10). Scale bar, 50 μm. ** *P* < 0.01

To further evaluate the prognostic value of DDK complex members in HCC patients, immunohistochemical staining on TMA was carried out. Unfortunately, we didn’t find a suitable DBF4 antibody for the IHC application. Therefore, only CDC7 was evaluated. Based on the IHC staining results, HCC patients were divided into two groups with high (*n* = 71) or low (*n* = 39) CDC7 expression, and representative images of staining were shown in Fig. 4A. The association of CDC7 expression and the clinical characteristics was presented in Table 1, and accordingly, the expression of CDC7 was highly correlated with tumor size (*P* = 0.011) and TNM stage (*P* = 0.008). Consistent with the findings in TCGA dataset, univariate and multivariate analysis revealed that the expression of CDC7 was closely associated with OS (Table 2), and significantly longer OS was found in patients with lower CDC7 expression (*P* = 0.009; Fig. 4B).

**Table 1:** Correlations between CDC7 and clinicopathological features of HCC patients

Variables	Cases	CDC7 expression		*P*
		Low (*n* = 39)	High (*n* = 71)	
Age (yr)				0.801
< 50	49	18	31	
≥ 50	61	21	40	
Gender				0.822
Male	58	20	38	
Female	52	19	33	
Tumor size(cm)				0.011
≤ 5	47	23	24	
> 5	63	16	47	
AFP (ng/ml)				0.699
≤ 20	45	15	30	
> 20	65	24	41	
TNM stage				0.008
I/II	49	24	25	
III/IV	61	15	46	
HBsAg				0.474
Positive	67	22	45	
Negative	43	17	26	
Vascular invasion				0.733
Presence	56	19	37	
Absence	54	20	34	
Multiplicity				0.682
Single	48	16	32	
Multiple (≥ 2)	62	23	39

**Table 2:** Univariate and multivariate analysis of different prognostic variables influencing OS in HCC

Variables	*n*	Univariate analysis		Multivariate analysis model	
		HR (95% CI)	*p*	HR (95% CI)	*p*
Gender		0.536 (0.642–1.669)	0.424		
Male	58				
Female	52				
Age (year)		0.517 (0.508–1.604)	0.401		
< 50	49				
≥ 50	61				
Tumor size (cm)		0.637 (0.663–1.807)	0.019	0.544 (0.487–1.339)	0.011
≤ 5	47				
> 5	63				
AFP(ng/ml)		1.224 (0.403–1.353)	0.718		
≤ 20	45				
> 20	65				
HBsAg		0.663 (0.347–1.294)	0.667		
Positive	67				
Negative	43				
TNM stage		1.408 (0.574–1.397)	0.021	1.307(0.610–1.547)	0.028
I/II	49				
III/IV	61				
Multiplicity		0.573 (0.607–1.302)	0.691		
Single	48				
Multiple (≥ 2)	62				
Vascular Invasion		1.003 (0.579–1.861)	0.842		
Presence	56				
Absence	54				
CDC7 expression		1.324(0.477–1.863)	0.011	1.345 (0.518–1.745)	0.015
High	71				
Low	39				

### The potential role of DDK complex in HCC development

**Fig. 5:**
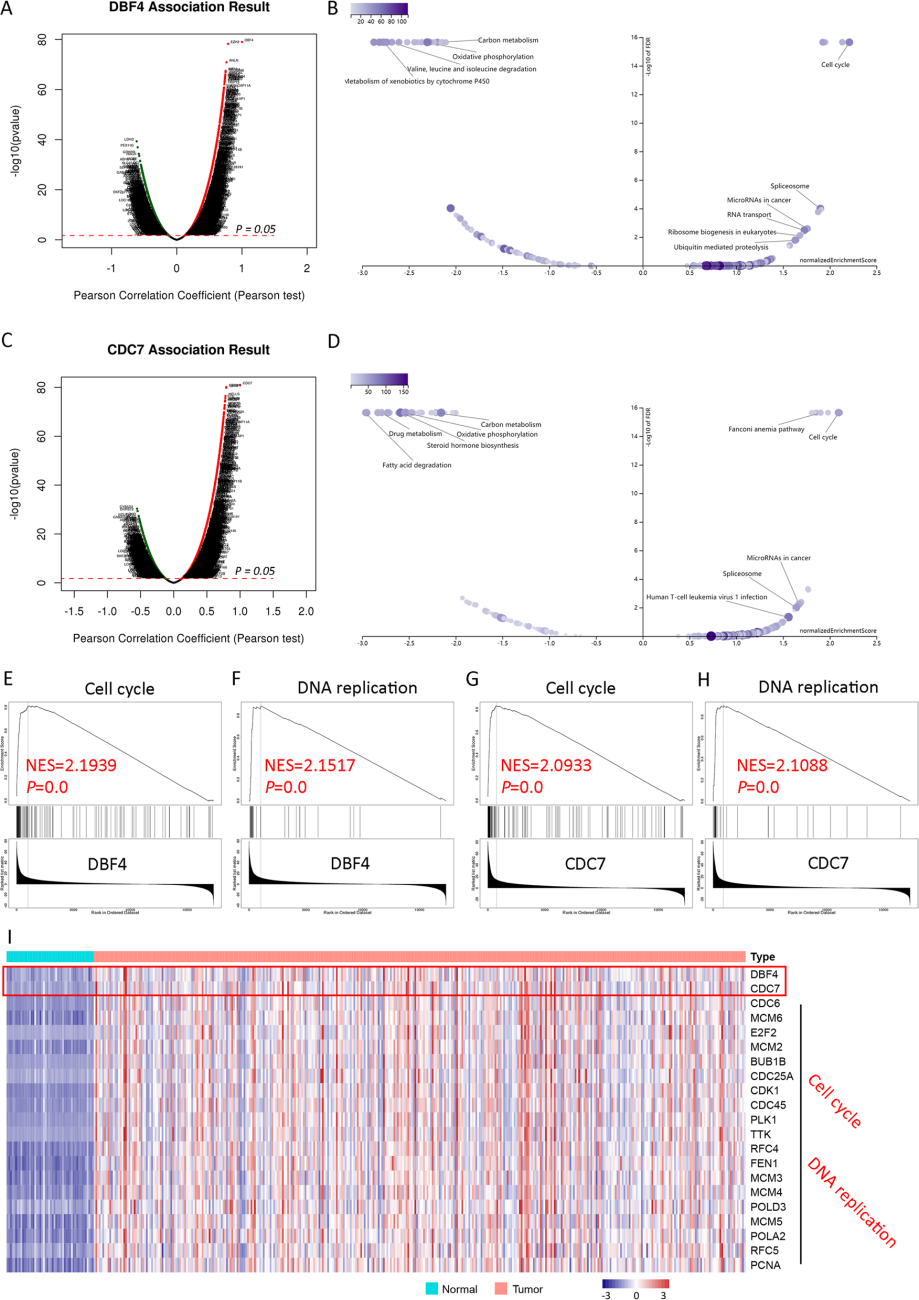
The pearson correlation and GSEA analysis results based on TCGA HCC dataset. **A** Volcano map showing the pearson correlation analysis result of DBF4 based on the gene expression profile of TCGA HCC dataset. **B** Volcano plot of GSEA analysis of DBF4. **C** Volcano map showing the pearson correlation analysis result of CDC7 based on the gene expression profile of TCGA HCC dataset. **D** Volcano plot of GSEA analysis of CDC7. **E–H** GSEA results of DBF4 and CDC7 on DNA replication and cell cycle, respectively. NES, normalized enrichment score. **I** Heatmap showing the most correlated genes with DBF4 and CDC7 in cell cycle and DNA replication

According to our results above, high expression of DDK complex may play a critical role in HCC development, while the underlying events remain unclear. As a critical regulator in DNA replication, we firstly analyzed the cell proliferation status in CDC7^High^ and CDC7^Low^ tumor samples. As demonstrated in Fig. 4C, more proliferating tumor cells were observed in CDC7^High^ tumor tissues. In order to further explore the possible correlated signal pathways through which DDK complex functions in the development of HCC, we firstly screened the genes that positively or negatively correlated with DBF4 or CDC7 in TCGA HCC data by pearson correlation analysis (Table S1 and S2), and then GSEA was performed on Linkedomics online website (http://www.linkedomics.org/) to identify the signaling pathways these genes belong to (Fig. 5A-D). According to the *P* value < 0.05, FDR < 0.05 and normalized enrichment score (NES), significant enrichment pathways were screened, and the top-ranked pathways were presented in Fig. 5B and D. The results demonstrated that genes were enriched in pathways facilitating tumor cell proliferation, especially in cell cycle and DNA replication (Fig. 5B, D and E–H), and the top-ranked associated genes were listed in Fig. 5I. These results suggested that highly expressed DDK complex members promotes DNA replication, thereby enhancing tumor cell proliferation, and that DDK complexes can promote HCC development in this way.

## Discussion

DNA replication, as one of the most fundamental processes for tumor cell proliferation, is sophisticated and finely regulated. Substantial evidence has proved that key regulators of DNA replication play critical roles in cell proliferation and are potential therapeutic targets for cancers [21, 22]. DNA replication requires the activities of CDKs and DDK complex, both of which are essential for DNA replication, chromosome segregation, centromeric heterochromatin formation, and genome maintenance of mitotic cells [23–25]. The role of CDKs, especially CDK4/CDK6 complex, in HCC development has been well illustrated, and its specific inhibitor has been adopted for HCC treatment [11–13]. However, the role of DDK complex in HCC remains unknown.

In this present study, we firstly comprehensively analyzed the expression of DBF4 and CDC7 in HCC patients, and found both of them were highly expressed in HCC tumor tissues. As a whole complex member and elevated in most cancer types, DBF4 and CDC7 were found positively correlated with each other in 26 TCGA cancer types, especially in HCC. In addition, their expression was found higher in advanced HCC patients with higher clinical stages and grades. Patients with higher DBF4 or CDC7 expression were more likely to have a poor prognosis. Finally, according to our HCC cohort, we found the expression of CDC7 was highly correlated with tumor size and TNM stage of HCC patients, and patients with higher CDC7 expression had a shorter overall survival.

In an unperturbed cell cycle, the DDK complex binds to and phosphorylates its essential target—the MCM2-7 ring to initiate DNA replication, which leads to gate opening between MCM2 and MCM5, allowing extrusion of single-stranded DNA generated by origin melting, which is critical for DNA replication. It has been reported that MCM family members may play important roles in HCC development and can serve as prognostic biomarkers in HCC [7–9], the function of which is largely dependent on DDK complex. Checkpoint is activated when cells suffer DNA damage or are depleted of dNTPs, in which cases DDK complex is phosphorylated by radiation-sensitive 53 (Rad53) kinase, causing its dissociation from chromatin [26, 27]. Therefore, the highly expressed DDK complex members in HCC may facilitate and accelerate tumor cell proliferation. This speculation was further confirmed by pearson and GSEA analysis, the genes highly associated with CDC7 and DBF4 are enriched in cell cycle, DNA replication and microRNA in cancer pathways.

Similar to CDKs, the DDK complex may serve as potential therapeutical target. Before that more studies are needed to explore the molecular mechanism underlying the elevation of their expression (upstream regulator, methylation, mutation or amplification, etc.) and the consequences caused by their ablation or silence, which may provide new insights and therapeutic cues for HCC patients.

## Conclusions

In summary, we performed a comprehensive analysis of the expression and potential role of DDK complex in HCC. Like most oncogenes, high expression of DBF4 and CDC7 is associated with poor prognosis, and both may be potential independent prognostic factors for HCC patients.

## Supplementary Information

**Additional file 1: Supplementary Fig. A.** the original western blot bands of DBF4, CDC7 and GAPDH of samples 1-7. **Supplementary Fig. B.** the original western blot bands of DBF4, CDC7 and GAPDH of samples 8-14.

**Additional file 2: Supplementary Table 1.** Genes highly correlated with CDC7 in TCGA HCC database.

**Additional file 3: Supplementary Table 2.** Genes highly correlated with DBF4 in TCGA HCC database.

## Data Availability

All data are available via the corresponding author.

## Abbreviations

HCC: Hepatocellular carcinoma
DDK: Dependent kinase complex
TMA: Tissue microarray
IHC: Immunohistochemistry
GSEA: Gene set enrichment analysis
OS: Overall survival
RFS: Recurrence free survival
DSS: Disease-specific survival
PFS: Progress-free survival
