# Supplementary material for: Analysis of the expression and prognostic significance of DDK complex in Hepatocarcinoma
Source: BMC Cancer. 2023 Jan 6;23:19. doi: 10.1186/s12885-022-10475-w (PMC9817372; doi:10.1186/s12885-022-10475-w)
Supplement: Supplementary file 1 — Additional file 1: Supplementary Fig. A. the original western blot bands of DBF4, CDC7 and GAPDH of samples 1-7. Supplementary Fig. B. the original western blot bands of DBF4, CDC7 and GAPDH of samples 8-14. [file 12885_2022_10475_MOESM1_ESM.docx]

**(A)**

CDC7

DBF4


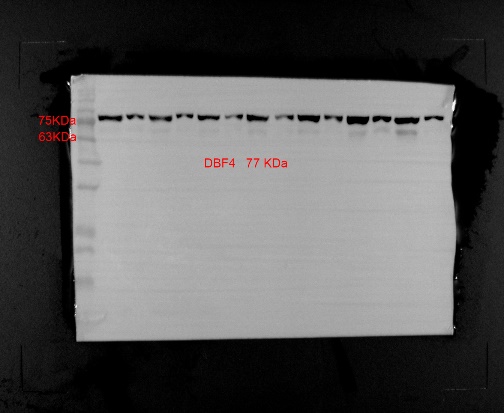

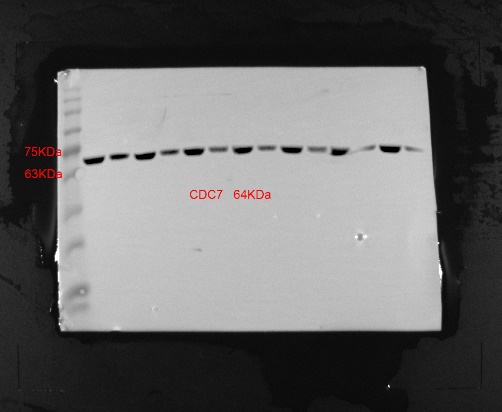


GAPDH


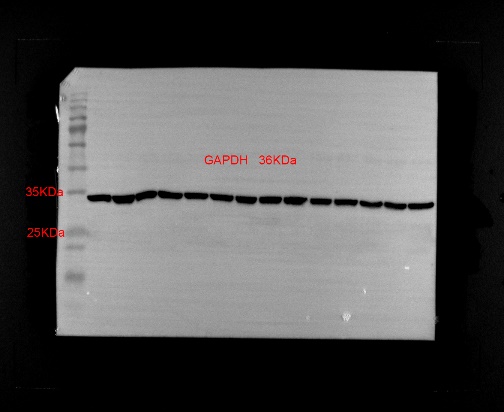


**Supplementary Fig. A the original western blot bands of DBF4, CDC7 and GAPDH of samples 1-7.**

**(B)**

DBF4

CDC7


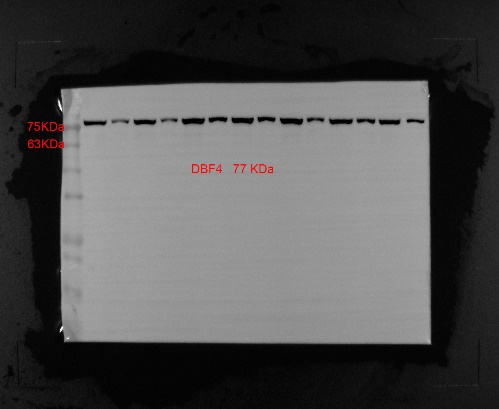

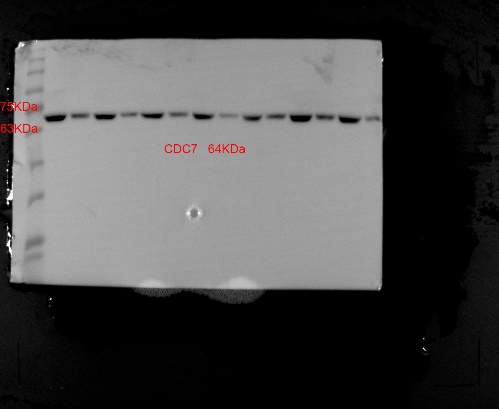


GAPDH


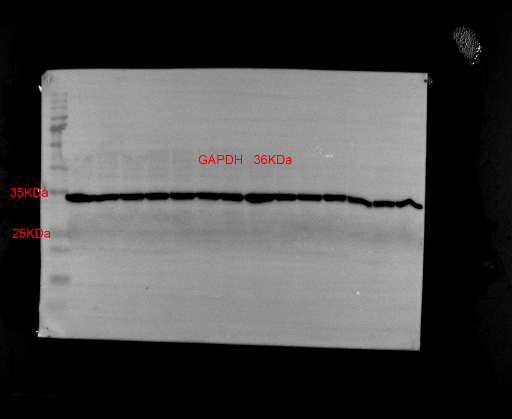


**Supplementary Fig. B the original western blot bands of DBF4, CDC7 and GAPDH of samples 8-14.**
